# Meta-Analysis Reveals That Explore-Exploit Decisions are Dissociable by Activation in the Dorsal Lateral Prefrontal Cortex and the Anterior Cingulate Cortex

**DOI:** 10.1101/2023.10.21.563317

**Published:** 2023-10-23

**Authors:** Daniel Sazhin, Abraham Dachs, David V. Smith

**Affiliations:** Department of Psychology & Neuroscience, Temple University, Philadelphia, PA, USA

**Keywords:** Exploration, Exploitation, Dynamic Decision Making, fMRI

## Abstract

Explore-exploit research has challenges in generalizability due to a limited theoretical basis of exploration and exploitation. Neuroimaging can help identify whether explore-exploit decisions use an opponent processing system to address this issue. Thus, we conducted a coordinate-based meta-analysis (N=23 studies) where we found activation in the dorsal lateral prefrontal cortex and anterior cingulate cortex during exploration versus exploitation, providing some evidence for opponent processing. However, the conjunction of explore-exploit decisions was associated with activation in the dorsal anterior cingulate cortex, dorsal medial prefrontal cortex, and anterior insula, suggesting that these brain regions do not engage in opponent processing. Further, exploratory analyses revealed heterogeneity in brain responses between task types during exploration and exploitation respectively. Coupled with results suggesting that activation in exploration and exploitation decisions is generally more similar than it is different suggests there remain significant challenges toward characterizing explore-exploit decision making. Nonetheless, dlPFC and ACC activation differentiate explore and exploit decisions and identifying these responses can help in targeted interventions aimed at manipulating these decisions.

## Introduction

Explore-exploit problems are ubiquitous in many real-world situations such as staying in one line of employment or moving to another, keeping versus selling a stock, or trying out a new ice cream flavor or sticking with what you know. Situations where a person does not have full knowledge of their opportunities and outcomes have a fundamental dilemma whether to explore the space of possibilities available to them or exploit what they already know. Due to their prevalence in naturalistic settings, explore-exploit dilemmas have been extensively investigated, with an emphasis on whether certain people are consistently likely to over or underexploit. Over and under exploitation is especially interesting in psychological research as markers of psychopathology, such as among people with anxiety, compulsivity and smoking habits (Aberg and Paz 2022; Merideth A. Addicott et al. 2014; L. S. Morris et al. 2016).

Despite the interest in explore-exploit tasks, generating generalizable insights from decisions made in the lab has several major challenges. The first is that explore-exploit situations generally contain many independent variables that are difficult to control, such as the hidden payoffs of existing options, the number of options available to the participant, the strategies guiding exploration (random or directed), and the time horizons of the tasks (Wilson et al. 2014). Even simply underlying the payoffs of these choices includes a multitude of decision variables such as risk, uncertainty, and ambiguity. Controlling these independent variables is necessary to assess if exploration and exploitation can be construed as a consistent and useful psychological construct. Second, is the lack of behavioral convergence across foraging and n-armed bandit tasks (von Helverson et al., 2019), which suggests that exploration and exploitation may not be guided by consistent attitudes. Third, there remains a lack of a unified theory of exploration and exploitation, behaviorally and neurally, as to whether exploration and exploitation are opponent processes, or resultant of the interaction of multiple underlying systems. These major questions suggest that reviewing the common features of exploration and exploitation could yield clarity theoretically and empirically as to how the field should understand these decisions. To do so, we first review extant literature, followed by conducting a co-ordinate based (CBMA) meta-analysis to understand which brain regions are consistently involved in exploration and exploitation.

### Understanding Explore-Exploit Decisions Behaviorally

Many tasks have been conceived to isolate explore-exploit decisions, though they mostly fall within two categories: foraging tasks (M. A. Addicott et al. 2017) and n-armed bandit tasks (Cohen, McClure, and Yu 2007; M. A. Addicott et al. 2017; Zhen et al. 2022). These tasks are highly prevalent in explore-exploit research because they have computationally optimal closed-form solutions. In foraging tasks, a participant selects whether to forage from a patch of resources such as an apple tree, or to travel to another patch at some distance from the current patch (Mobbs et al. 2018; Bernstein, Kacelnik, and Krebs 1988; Stephens and Krebs 1986). The optimal strategy is determined by a marginal value theorem (pMVT) which is determined by the payoffs within a current patch and the distance of the next patch (Charnov 1976). In n-armed bandits tasks, the participant decides which slot machine they would like to sample from (Bellman and Kalaba 1957). Explore-exploit decisions are classified through a variety of computational algorithms, such as Boltzmann exploration (softmax), algorithms, and reinforcement learning (Kuleshov and Precup 2000). Ultimately, when the participant chooses bandits with high expected value, the decisions are classified as exploitative and when they choose bandits with lower or unknown expected value, they are classified as explorative (Daw et al., 2006).

While there are canonical foraging and n-armed bandit tasks, there are many other variants of these tasks. One variation of the n-armed bandit task is the Horizon Task which runs for 15, 30, or 45 minutes was developed to discern if task length affects behavior (Trudel et al. 2020). Another variation of the n-armed bandit is the Leapfrog task where two bandits’ values are fixed until the lower value bandit ‘leapfrogs’ over the higher value bandit at random intervals (Blanco et al. 2016). Variations of foraging also include the Clock Task (Moustafa et al. 2008). Optimal stopping problems such as the Secretary Task (Freeman 1983) are also sometimes grouped as explore-exploit dilemmas. With such a variety of tasks, a critical question is whether the independent variables manipulated within these tasks guide exploration and exploitation, or if general tastes in exploration and exploitation tend to guide behavior. If choices are inconsistent between tasks, then exploration and exploitation should not be conceived as independent constructs, but rather the interaction of the underlying independent variables. Recent evidence suggests that foraging tasks and n-armed bandits lack behavioral convergence (von Helversen et al. 2018) which suggests that how people exploit and exploit in n-armed bandits does not predict how people will explore or exploit in a foraging task. The lack of behavioral convergence between tasks is a major challenge as this suggests that there is a lack of a unifying psychological mechanism underlying exploration and exploitation decisions.

Another approach may be to assess if economic or psychological differences can reliably differences in exploration or exploitation. In the context of temporal discounting problems there have been mixed findings, with one investigation finding associations between temporal discounting and directed exploration and no relationship between temporal discounting and random exploration (Sadeghiyeh et al. 2020) and another study suggesting inconsistent preferences to temporal discounting and exploration and exploitation across multiple studies (Meyers and Koehler 2021). In assessing effects of impulsive behaviors, or risk attitudes, there were no significant associations with foraging decisions though gamblers exhibited more exploratory behavior (Merideth A. Addicott et al. 2015).

Other kinds of individual difference measures have yielded somewhat more robust associations with exploratory or exploitative behaviors. Experiences of lifetime scarcity was related to decreased resource-maximizing decision-making (Chang, Jara-Ettinger, and Baskin-Sommers 2022) and individuals with adverse childhood experiences explore less in a foraging task (Lloyd, McKay, and Furl 2022). Contextual effects in foraging tasks affect the explore-exploit tradeoff, with greater acute stress yielding overexploitation (Lenow et al. 2017), increased in arousal associated with increased levels of exploration, and increases in valence substantially increased exploitation (van Dooren et al. 2021). Further, there are associations between psychopathologies and explore-exploit decisions. Some examples include that that smokers make less initial exploratory choices (Merideth A. Addicott et al. 2012), people with greater anxiety and depression use lower levels of directed exploration (Smith et al. 2021), subjects with alcohol use disorders or binge eating disorders had decreased exploration when confronted with losses (L. S. Morris et al. 2016), and people with schizophrenia overused random exploration strategies (Speers and Bilkey 2023). Taken together, explore-exploit tasks have been applied in a variety of psychological domains, yielding little consistency in terms of economic decisions, though an attenuated ability to optimize these decisions if people have maladaptive psychological or psychiatric attributes.

### Neurobiological Mechanisms of Exploration and Exploitation

**Figure 1:**
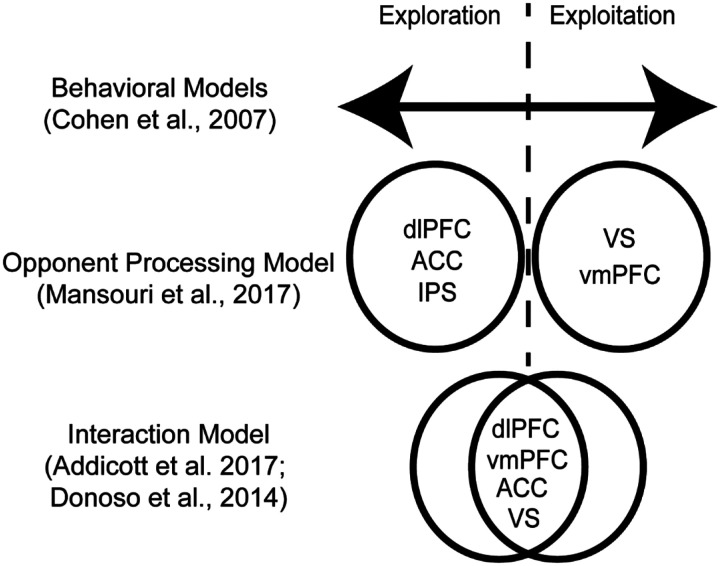
Models of exploration and exploitation. Behavioral models of explore-exploit decisions envision an optimal set of decisions, with people expressing tendencies to over or underexploit on a continuum of possible options. Nonetheless, the underlying mechanisms may be a binary or opponent processing model, where people are either exploring or exploiting, with corresponding brain activation. Another model suggests that exploration and exploitation is more of an interaction model, where all of the brain regions are involved in both exploration and exploitation, though may interact more or less depending on the explore-exploit situation.

Explore-exploit tasks lack behavioral convergence, contain a multitude of possible independent variables, and lack a coherent theory as to whether exploration and exploitation are a product of disparate versus unified mechanisms. With the lack of clarity as to the constructs and behaviors guiding explore-exploit decisions, another approach could examine the neurobiological factors that are consistent across explore-exploit choices. One notable challenge that could be observed neurobiologically is if explore-exploit tasks elicit a consistent or disparate set of responses during exploration versus exploitation (Cohen, McClure, and Yu 2007) (*see* figure 1). If exploration and exploitation elicit reliably different patterns of activation across various tasks, it could provide a window into what mechanisms may modulate explore-exploit decisions through an opponent processing system. Over the past two decades, there have been enough neuroimaging studies conducted in explore-exploit tasks that reviewing these common patterns may provide insight into explore-exploit decision making as a whole.

Explore-exploit decisions in neuroscience have isolated several key cortical and subcortical brain regions that contribute to these choices (Dennison, Sazhin, and Smith 2022). In animal literature, the Anterior Cingulate Cortex (ACC) has been identified as a major modulator of explore-exploit decisions. Versions of the n-armed bandits have been fitted for rats and mice with the use of n-armed radial mazes (Ohta et al. 2021). Anterior Cingulate Cortex (ACC) activation has been linked to foraging in rats in an adapted patch foraging task (Kane et al. 2022) and a two-armed bandit monkey lesion study (Kennerley et al. 2006). Similarly, the dACC is tied to both exploration and exploitation in a monkey foraging task (Ramakrishnan, Hayden, and Platt 2019; Hayden, Pearson, and Platt 2011). Nonetheless, other findings suggest that the VS and amygdala represent immediate and future value of exploratory choices respectively in rhesus monkeys (Costa, Mitz, and Averbeck 2019). In human neuroimaging studies, there are some commonly cited areas of activation in brain regions associated with cognitive control (dlPFC), reward (VS), and attention (ACC), which are commonly used in region of interest (ROI) analyses (Shenhav et al. 2014; Wiehler, Chakroun, and Peters 2021).

### Evidence for Models of Exploration and Exploitation

While it is known that an array of brain regions are involved in explore-exploit decisions, it remains a key challenge to understand how these brain regions respond to dynamic environments. Two major accounts explain explore–exploit behaviors, which are the interaction of several neural regions depending on contextual features of the explore-exploit decision (e.g., dACC, dorsal striatum, lateral PFC, and VS; Donoso et al., 2014), or a dual-system driven by opponent processes of exploration and exploitation in frontoparietal regions (e.g., dlPFC, ACC, IPS vmPFC and VS; Mansouri et al. 2017) (*see* figure 1). If exploration and exploitation can be construed as opponent processes, this could allow for targeted interventions aimed at modulating these behaviors. Recent findings suggest support for both accounts, with frontopolar regions such as the dlPFC orienting switching decisions and the Intraparietal Sulcus (IPS) directing attention (M. A. Addicott et al. 2017).

In trying to reconcile these accounts, studies tease apart how certain elements of explore-exploit dilemmas contribute to those decisions. For instance, understanding how environmental uncertainty and trends in information mediates this process may inform some of the underlying mechanisms in explore-exploit choices, with environmental uncertainty of new options (Badre et al. 2012; Navarro, Newell, and Schulze 2016; Tomov et al. 2020) seemingly largely processed in the PFC. Uncertainty in an environment has been represented in the brain in several ways, with relative uncertainty in the right rostrolateral PFC (Badre et al. 2012) and striatal dopamine function (Frank et al. 2009) driving directed exploration. The vmPFC was implicated in representing environmental uncertainty (Trudel et al. 2020), evidence accumulation in switching decisions (Blanchard and Gershman 2018), and determining the value of well-defined foraging options (Kolling et al., 2012). These findings taken together reinforce the importance of both frontopolar and subcortical regions in explore-exploit decisions, though it remains unclear to what degree an opponent process model driven by the frontoparietal cortex is supported by the weight of the evidence.

In sum, the current state of knowledge is limited in identifying consistent elements supporting neural circuitry associated with explore-exploit decisions, whether there are systematic biases in the literature, and if certain brain regions remain underemphasized in the reporting and interpretation of the data. One means of addressing these limitations is through quantitatively assessing patterns of activation across neuroimaging studies using coordinate-based meta-analyses (CBMA). We hypothesized that there will be convergence across explore-exploit studies in activation in the vmPFC, dlPFC, VS, ACC, and IPS while making explore-exploit decisions. These decisions are differentiated from the feedback phase where participants receive rewards based on their decision. The feedback phase does not constitute a strategic decision to explore or exploit based on preceding information. Next, we expected that the exploitation versus exploration decision would be associated with greater vmPFC, VS, ACC activity than the exploration phase and that the exploration phase will be associated with greater activation in the IPS and dlPFC than in the exploitation phase.

Since we began this investigation, one group of researchers has conducted a meta-analysis of explore decisions (Zhen et al. 2022) finding that exploration results in consistent activation in the dorsal medial prefrontal cortex and anterior insula, dorsolateral prefrontal cortex inferior frontal gyrus, and motor processing regions. We extend on their results by comparing exploration versus exploitation and exploring task-based differences between exploration and exploitation. These comparisons help identify activation which is unique to these decisions and differentiates exploration from exploitation, thereby helping identify to what degree we can theoretically understand these choices arising from an opponent processing model. We also explore activation differences between n-armed bandits and other types of explore-exploit tasks while making exploration or exploitation decisions. In sum, we investigate the common patterns of activation across explore-exploit tasks, whether there are systematic biases in the literature, and if there are other regions that are underemphasized in the interpretation of the data.

## Materials and Methods

### Inclusion Criteria and Study Selection

The current coordinate-based meta-analysis primarily followed PRISMA guidelines for meta-analyses for inclusion criteria, filtering, and analyses (Moher et al. 2009). We incorporated a pre-registration (https://aspredicted.org/7hc7c.pdf), which detailed the hypotheses and analyses we intended to use. We conducted a systematic literature search to identify explore-exploit studies that used neuroimaging techniques. First, we identified search terms through identifying task names in several existing explore-exploit literature reviews (Cohen, McClure, and Yu 2007; M. A. Addicott et al. 2017; Zhen et al. 2022). Potentially eligible studies published through 1/01/2023 were identified by searching the PUBMED using the grouped terms: (n-armed OR exploration-exploitation OR explore-exploit OR multi-armed OR forage OR foraging OR “reward rate” OR (explore AND exploit) OR “reward trend” OR “clock task” OR clock-task OR “temporal-difference” OR “patch leaving” OR patch-leaving OR leave-stay OR “time horizon” OR “horizon task” OR bandit OR MVT OR “marginal value theorem” OR leapfrog OR “leap frog” OR leap-frog OR prey model OR “diet breadth model” OR “web surfing task” OR “web-surfing task” OR trend-guided OR “uncertainty driven”) AND (fMRI OR “functional magnetic resonance imaging” OR neuroimaging OR brain OR neural OR MNI OR “Montreal Neurological Institute” OR Tal OR coordinates). To enhance search sensitivity, the reference lists of the retrieved articles and review papers were further checked to identify potentially relevant articles. Further, we included studies that reported whole-brain analyses, as region of interest based analyses can bias coordinate-based meta-analyses (Moher et al. 2009) and were excluded. Finally, we incorporated studies that reported coordinates in a standard stereotactic space [ie: Talairach or Montreal Neurological Institute (MNI) space]. The search process was conducted by Avi Dachs, with the first author identifying the studies accepted for final inclusion in the meta-analysis. For eligible studies that did not report whole-brain data, we contacted authors if the required information was unavailable in the published reports.

**Figure 2:**
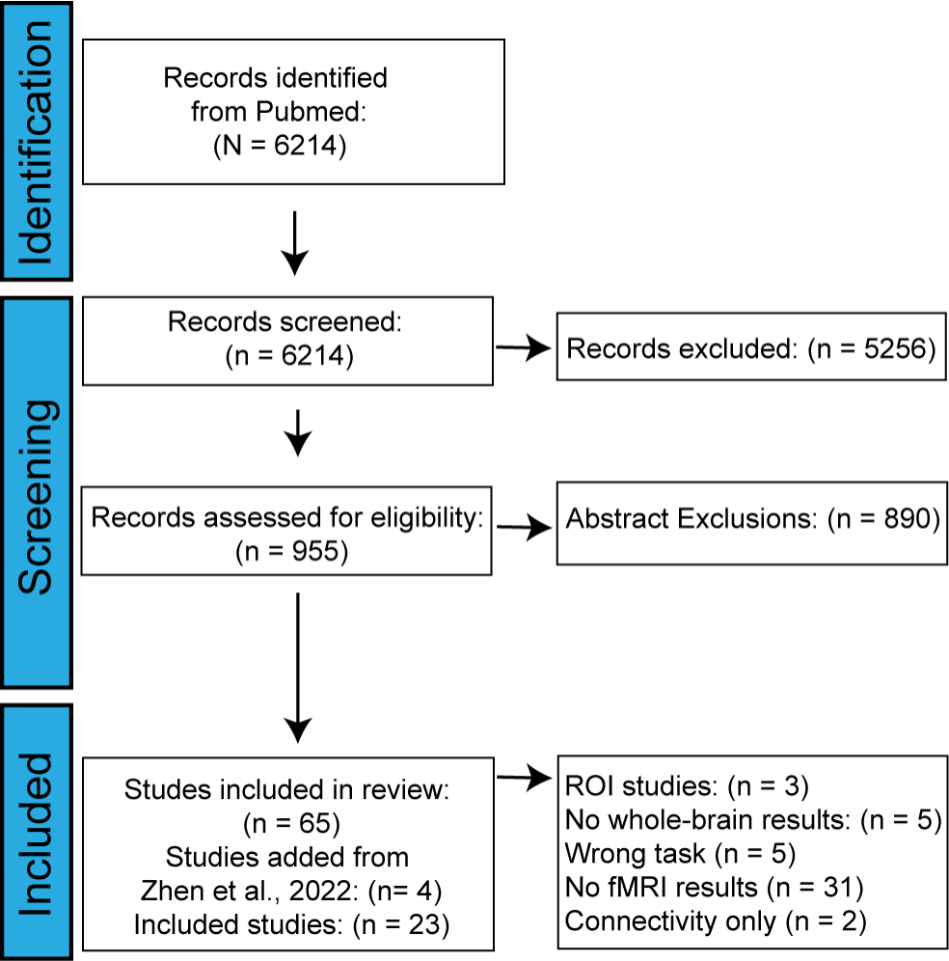
Inclusion criteria. Using the PRISMA flowchart system, we report the studies we identified, screened, and included in the meta-analysis. After we completed our initial search, four more studies identified by another meta-analysis (Zhen et al., 2022) that assessed activation during exploration. Starting from an initial search of 6,214 studies, we included n=23 studies in our analyses.

The initial PUBMED search yielded 6,214 papers. 5,256 papers were then excluded based on title, leaving 958 papers to be excluded by the abstract and the full text’s contents. Of the 958 remaining papers, 762 papers were excluded for not covering explore and exploit tasks, 72 relevant papers were excluded for not collecting fMRI data, 45 animal studies were excluded, and 14 non-empirical papers were excluded, leaving only 65 papers for data extraction and coding (see Figure 2). In the coding phase, 47 more papers were excluded due to data that were incompatible with our analysis (i.e., not fmri or whole-brain), leaving a total yield of 19 papers. Finally, our list of papers was cross-referenced with the papers included in a similar meta-analysis (Zhen, 2022) revealing 4 papers that were wrongly excluded by our search. After these papers were added, our final corpus includes 23 papers with a cumulative N of 602 participants (see Figure 2 and Table 1). In total, we accumulated 13 number of n-armed bandit studies, which ranged in various numbers of bandits presented to the participant. We found 7 foraging tasks and 3 other tasks, including a problem-solving task, clock hand task, web surf task, and an observe-bet task. We grouped non-n-armed bandit tasks into an “other” category with a total of 10 studies to serve as a comparison group.

### Statistical Analysis

We conducted a CBMA meta-analysis using Seed-based d mapping software (SDM-PSI version 6.22). SDM was implemented using several steps to conduct analyses described in the SDM tutorial and manual. First, we imported the screened data by preparing folders with MNI text files that reported the clusters and t values for each coordinate. Exploration and exploitation decisions were grouped based on the constructs reported in each study. 12 studies reported explore>exploit and exploit>explore contrasts and were coded as exploration and exploitation respectively (see Table 2). Other studies reported parametric effects for exploration and exploitation decisions (see Table 2). Specifically, one study coded foraging value-decision value contrast for exploration and search value- decision value contrast for exploration (Kolling et al. 2012). We classified reinforcement learning associated with experience as exploration (Dunne, D’Souza, and O’Doherty 2016). Switch-in events were classified as exploration and activation associated with confirmation and rejections events as exploitation due to the behavioral design (Donoso, Collins, and Koechlin 2014). Studies modulated uncertainty (Badre et al. 2012; Trudel et al. 2020), relative value (Howard-Jones et al. 2010), task difficulty (Abram et al. 2019), search evidence, and search cost (Zacharopoulos et al. 2018). Others modulated exploration or exploitation through reinforcement learning algorithms (Wittmann et al. 2016), presence or absence of newly available information (Wang and Voss 2014), and the advantageousness of the environment (Mobbs et al. 2013).

Next, we created an SDM table with all the respective peak coordinates. We noted t-stats in the SDM table with respect to effect sizes and converted reported p and z stats using the SDM “convert peaks” function (see Table 2). Then, we completed preprocessing using Functional MRI as its modality, with a gray matter correlation matter template following validated methods (Albajes-Eizagirre et al. 2019; J. Radua et al. 2012). We used a 1.0 anistropy setting, a 20 mm FWHM isotropic kernel, a gray matter mask, and a standard 2mm voxel size. This was followed by a mean analyses with 50 imputations (Joaquim Radua and Mataix-Cols 2009). To compare exploration and exploitation decisions, and n-armed bandit versus other tasks, we generated linear models respectively where we compared these groups by assigning a linear model analysis (Joaquim Radua et al. 2010). We used the SDM meta-regression tool with prediction dummy variable {exploit=1, explore=0} and {n-armed=1, other=0} for the positive side of the significance test. Next, we preformed family wise error corrections and using n=1000 permutations (Albajes-Eizagirre et al. 2019). The results were thresholded using TFCE with a probability of P<0.05. Masks were created and their values were extracted for reporting. For the conjunction of explore and exploit conditions, we conducted a CBMA of explore and exploit conditions respectively, and then used the multimodal function provided by SDM to produce the conjunction map.

To assess potential heterogeneity and potential bias in the CBMA results, we extracted funnel plots. We report the strength of evidence through multiple robustness considerations, study heterogeneity (I2 statistic), effect of small studies on the results (metabias) with resulting funnel plot asymmetry, and excess significance. The funnel plots are constructed through assessing the residual, or the weight each study has in the meta-analysis, with the size of its treatment effect, identified as precision on the y axis, though these tests must be interpreted with caution as publication bias can arise from multiple sources (Sterne et al. 2011). All analyses were completed in Montreal Neurological Institute (MNI) space. To report consistent results across human brains (Mazziotta et al. 1995), we show probabilistic anatomical labels for clusters of activation using the Harvard–Oxford cortical and subcortical atlases (Desikan et al. 2006).

## Results

**Figure 3:**
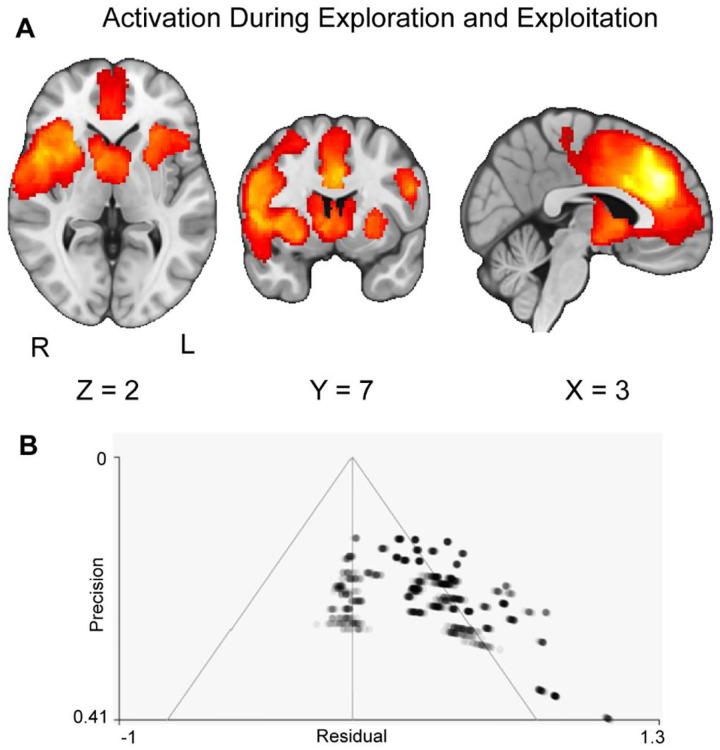
Clusters of activation (red) across all explore-exploit tasks, in both the exploration and exploitation conditions. Panel A shows that our results are consistent with our hypotheses, showing activation in the ACC, IPS, dlPFC, vmPFC, and VS. Maps were thresholded using TFCE at P=.05 and rendered in MRIcroGL. The map is rendered in radiological view. Panel B shows the funnel plot associated with the first cluster in our results. Each gray dot is a study, with darker dots indicating more results in an area of the plot. The funnel plot was generated through multiple imputations, yielding more dots than studies included. Overall, the plot indicates possible publication bias in the results. Thresholded and unthresholded images are available at: https://neurovault.org/images/804127/ and https://neurovault.org/images/804137/.

We completed three meta-analyses that assessed activation across explore-exploit tasks, followed by activation specific to exploration and exploitation. First, we assessed the convergence across explore-exploit studies. This omnibus analysis would reveal common patterns of activation specific to making decisions in explore-exploit situations. We hypothesized that there will be convergence across explore-exploit studies in activation in the vmPFC, dlPFC, VS, ACC, and IPS. Our results indicate two significant clusters of activation in the left superior frontal gyrus, right nucleus accumbens (NAcc) (see Figure 3, Table 3). Using the Harvard-Oxford Atlas, these clusters encompass the NAcc, paracingulate gyrus, and the Angular gyrus extending into the Supramarginal gyrus, providing meta-analytic evidence supporting these regions as centers of activation in explore-exploit situations. We followed up with analyses of metabias and excess significance, finding that first cluster reported excess significance (P<.001) suggesting the observed number of significant findings is greater than the number of significant findings expected given the power of the tests (Ioannidis and Trikalinos 2007). The first cluster may have metabias effects (p=.029), which is inflated effect sizes resultant from small studies.

### Neural Responses Between Exploration versus Exploitation Phases

**Figure 4.**
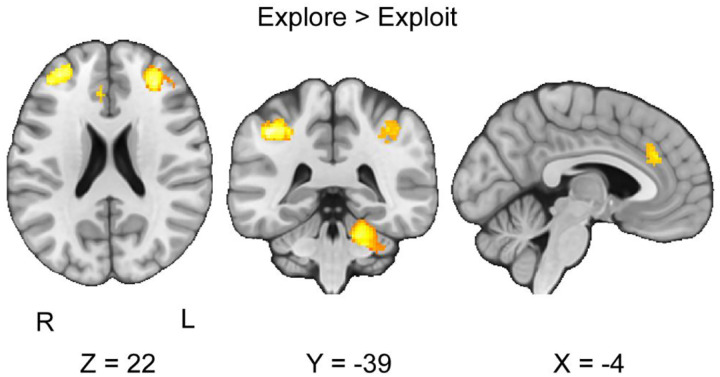
Clusters of activation in the contrast between explore versus exploit phases. Our results indicate that during the exploration versus exploitation phases, we find stronger activation in the dlPFC, ACC, and cerebellum. Maps were thresholded using TFCE at P=.05 and rendered in MRIcroGL. The map is rendered in radiological view. Thresholded (https://neurovault.org/images/804131/) and unthresholded (https://neurovault.org/images/804138/) images are available at Neurovault.

We conducted a CBMA contrasting the exploration and exploitation conditions across all the explore-exploit tasks. We hypothesized that the exploitation phase would be associated with greater vmPFC, VS, ACC activity than the exploration phase. Our analyses did not find any significant clusters of activation in the contrast between the exploit versus explore phases. Next, we hypothesized that the exploration phase would be associated with greater activation in the IPS and dlPFC than in the exploitation phase. Our results indicated six significant clusters of activation in the cerebellum, left and right dorsolateral prefrontal cortex, and left anterior cingulate cortex (*see* Figure 4, Table 3). Using the Harvard-Oxford Atlas, our results were consistent with our hypotheses in finding stronger activation in the dlPFC during exploration versus exploitation. We followed up with analyses of metabias and excess significance, finding no significant metabias or excess significance for this CBMA.

### Neural Responses to the Conjunction Between Exploration and Exploitation Phases

**Figure 5.**
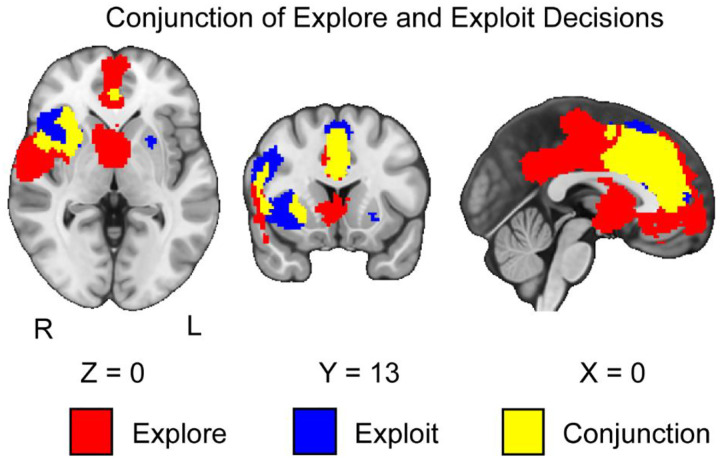
Clusters of activation in the conjunction of explore and exploit decisions. Our results indicate that during both explore and exploit phases, there is activation in the ACC, dmPFC, and AI. Maps were thresholded using TFCE at P=.05 and rendered in MRIcroGL. Red corresponds to activation during exploration, blue during exploitation, and yellow for the conjunction between both decisions. The map is rendered in radiological view. Thresholded images are available at https://neurovault.org/images/804128/, https://neurovault.org/images/804129/, and https://neurovault.org/images/804130/. Unthresholded images are available at https://neurovault.org/images/804142/, https://neurovault.org/images/804143/, and https://neurovault.org/images/804144/.

We conducted a conjunction analysis across exploration and exploitation in the sample of studies we collected. We hypothesized that the conjunction of explore and exploit phases would be associated with activation in the IPS, dACC, and dlPFC. Supporting our hypothesis, we found common activation in the dACC in the conjunction between exploration and exploitation decisions (*see* Figure 5). In contrast with our hypothesis, we did not find convergence in the IPS or dlPFC. We also found conjunctive patterns of activation in the dmPFC and anterior insula.

### Differential Activation Between N-Armed versus Other tasks During Exploration and Exploitation

**Figure 6.**
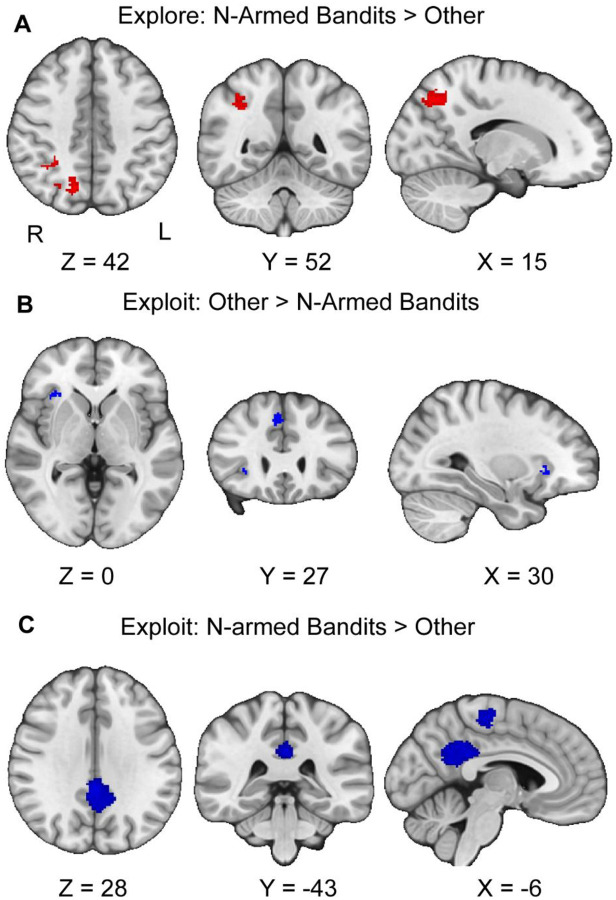
Clusters of activation during exploration and exploitation, between n-armed bandits and other tasks. Our results indicate that there are differences in activation between tasks during exploration and exploitation. Red activation corresponds to exploration and Blue to exploitation. In panel A, we report the contrast between n-armed bandits and other tasks during exploration. We find activation in the left and right somatosensory cortices and visual areas. In Panel B, during exploitation, we find that in the contrast between other versus n-armed bandits that there are differences in activation in the insula and dmPFC. In Panel C, we report that during exploitation, there are activation differences between n-armed bandits versus other tasks in the posterior cingulate cortex, and in the left and right motor areas. Maps were thresholded using TFCE at P=.05 and rendered in MRIcroGL.The map is rendered in radiological view. Thresholded images are available at Neurovault: https://neurovault.org/images/804132/, https://neurovault.org/images/804133/, and https://neurovault.org/images/804134/. Unthresholded images are available at Neurovault: https://neurovault.org/images/804139/, https://neurovault.org/images/804140/, and https://neurovault.org/images/804141/.

We followed up our pre-registered hypotheses by assessing if there are differences between activation in n-armed bandit tasks compared to other tasks during exploration and exploitation. If there are activation differences between tasks, this may suggest that these tasks are not eliciting consistent patterns of activation in exploration and exploitation as may be expected. During the exploration phase, we found that other tasks versus n-armed bandits resulted in three significant clusters, in the right precuneus, and the left and right supplementary motor areas (see Table 3, figure 6). We found no significant meta bias or excess significance in these results. There were also no significant results for the contrast between n-armed bandits versus other tasks.

Next, we followed up with assessing differences between n-armed bandit versus other tasks during the exploitation phase. During exploitation, the contrast between n-armed bandits versus other tasks yielded four significant clusters in the right rolandic operculum, left temporal gyrus, left median cingulate, and left paracentral gyrus (see Table 3, Figure 5). None of these clusters reported any metabias or excess significance. For the contrast between other tasks versus n-armed bandits during exploitation, we found two significant clusters in the right superior frontal gyrus and right insula (see Table 3, Figure 5). The first cluster reported possible excess significance in the data (p<.001), though no metabias. The funnel plot did not visually suggest that there was bias in the results.

## Discussion

This investigation conducted a coordinate-based meta-analysis of explore-exploit tasks. We included n-armed bandit and other types of explore-exploit tasks and analyzed them to assess patterns of activation consistent across explore-exploit decisions, unique to exploration and exploitation decisions respectively, and activation differences between tasks. First, we found consistent activation unique to exploration and exploitation decisions, with activation in the dlPFC, vmPFC, ACC, IPS, dmPFC, and VS. Second, we found stronger activation in the dlPFC and dACC during exploration versus exploitation. We found no differences between exploitation versus exploitation. Thirdly, we conducted an exploratory analysis assessing differences between n-armed bandits and foraging tasks during exploration and exploitation respectively. During exploitation, we found differences in activation in the PCC between n-armed bandits and other tasks. During exploitation, we found differences in activation in the AI and ACC between other tasks and n-armed bandits.

Overall, our meta-analytic results support past findings that have identified critical regions involved in exploration and exploitation. Specifically, we found convergence in brain regions identified in the seminal study by Daw et al., 2006 with activation in the dlPFC, vmPFC, IPS, ACC, and VS unique to explore and exploit decisions. Nonetheless, this result may be tempered as the first cluster identified may have publication bias and report the presence of more significant findings than would be expected given the power of the tests. Further, the main body of the significant effects may be inflated due to effect sizes resultant from the incorporation of studies with small sample sizes. It is difficult to conclude whether there is actual publication bias as there are several possible explanations for this effect, including true heterogeneity within the effects measured (Sterne et al. 2011). One interpretation is that exploration and exploitation may incorporate other brain regions, or that the regions identified may have somewhat inflated effect sizes.

### Opponent Processing versus Interactive Models of Exploration and Exploitation

When we investigated the contrast between exploration and exploitation, we found stronger dlPFC and ACC activation during exploitation versus exploration, suggesting that these regions are part of opponent processes in explore-exploit decisions. These results are consistent with past findings (Laureiro-Martínez et al. 2014), suggesting that the dlPFC may contribute to tracking the value of alternative choices (Laureiro-Martínez et al. 2015; Raja Beharelle et al. 2015), attend to risk (Obeso et al. 2021), track uncertainty (Chakroun et al. 2020; Tomov et al. 2020), and guide directed exploration (Zajkowski, Kossut, and Wilson 2017). The ACC may contribute to exploration versus exploitation by tracking trends in foraging tasks (Wittmann et al. 2016; Kolling et al. 2012), preparation of movement away from disadvantageous foraging patches (Mobbs et al. 2013), with more self-focused individuals showing lower activity in dACC compared to individuals who were foraging for others (Zacharopoulos et al. 2018), and the evaluation of salient feedback for learning optimal strategies (Amiez et al. 2012). Nonetheless, the interpretations emphasizing the role of the ACC in foraging may be confounded as one investigation found that dACC engagement was only explained by choice difficulty, and not the value of foraging (Shenhav et al. 2014). Integrating the dlPFC and ACC roles in regulating exploration versus exploitation is consistent with recent findings suggesting that these regions could be part of a circuit that modulates strategic decisions (Jahn et al. 2023) and contribute toward the opponent processing of exploration or exploitation.

However, there remain two large issues in interpreting exploration and exploitation through the lens of the opponent process model. The first is that there are more similarities than differences in activation across our results. Even when controlling for the effects of exploration and exploitation decisions specifically, our conjunction analyses reveal that exploration and exploitation generally elicit similar patterns of activation, particularly in the ACC, dmPFC, and AI. Extending on a previous meta-analysis suggests that these regions are not unique to exploration (Zhen et al. 2022), but are also involved in exploitation. As a result, when differences are reported in these regions, they may be due to the interaction of more complex underlying variables modulating these brain processes rather than a product of a general opponent processing system for exploration versus exploitation decisions.

Secondly, our exploratory analyses suggest that there remains substantial heterogeneity between tasks. This issue may speak to the lack of behavioral convergent validity between these tasks (von Helversen et al. 2018), which is to say that a participant exploiting in a foraging task does not predict how they will exploit in an n-armed bandit task. During exploitation, we found differences in activation in the insula and dmPFC between n-armed bandits and other tasks. Further, our meta-analysis revealed that exploiting while using n-armed bandit tasks compared to other tasks was associated with deactivation in the posterior cingulate cortex (PCC). In theory, if exploration and exploitation reliably elicit similar responses, we could expect to not see differences between these tasks. Nonetheless, the differences in AI, PCC, and ACC activation can be modulated by many possible cognitive processes such as emotion (Rolls 2019; Zhang et al. 2020) and attention (Maddock, Garrett, and Buonocore 2002; Pearson et al. 2011). These results suggest that while the dACC and dlPFC differentiate exploration and exploitation, these constructs remain fragile to the context of the decision based on task, and that most of the activation associated with these decision processes is indistinguishable and is modulated based on context. As such, the interactive model of exploration and exploitation is generally a better descriptor of these constructs, though the dlPFC and ACC can act as opponent processes between these types of decisions.

### Limitations

Although our work has found that exploration and exploitation can be dissociated by dlPFC and ACC activation, we acknowledge our study has several notable limitations. First, while we have N=23 studies, this quantity is fairly low for a CBMA type meta-analysis, which has a common benchmark suggests a minimum of 17–20 Experiments (Yeung et al. 2019). However, the exploratory CBMA of n-armed bandits versus other tasks during exploration and exploitation contrasted 13 versus 10 studies. As this sample size is below the benchmark, it should be considered exploratory. Nonetheless, there is a lack of clear guidance as to what are acceptable sample sizes using SDM, as they are highly contingent on the effects measured, number of participants, and whether thresholded images are included or not. Second, with the relatively small selection of studies, the inclusion of covariates such as age, sex distribution, and imaging parameters were not included. As more studies are completed, future researchers can assess the effects of these covariates.

Other limitations extend beyond meta-analytic methods in assessing exploration and exploitation more generally. Explore-exploit tasks limit the manner information is presented, and a latent variable that may bias switching decisions includes the trend in information. Some studies have started to explore the effects of trends in information (Wittmann et al. 2016; Kolling et al. 2012; Vestergaard and Schultz 2020), though it remains underexplored how trends bias people to act too soon or too late. Further, brain connectivity (Friston 2011; Utevsky et al. 2017) may reveal patterns of explore-exploit decision making, yet few connectivity studies (S. Morris et al. 2015; Tardiff et al. 2021) have been completed in this domain. Since the default mode network (DMN) is implicated in executive function and cognitive control (Fox et al. 2005), and the executive control network (ECN) serves to rapidly instantiate new task states (Marek and Dosenbach 2018), both the DMN and ECN may interact to drive exploiting versus exploring decisions. Future studies may reconcile the gap that remains in understanding how explore-exploit decisions are associated with brain connectivity patterns.

While acknowledging limitations for generalizing both behavioral and neural results resulting from exploration and exploitation, finding that the dlPFC and ACC reliably distinguish exploration and exploitation could fuel important future directions. First, a fruitful future direction includes modulating dlPFC responses, which are quite common in transcranial stimulation work. Since there are many links between dlPFC and psychopathology such as schizophrenia (Wu et al. 2017), anxiety (Balderston et al. 2020), and substance use (Goldstein and Volkow 2011), regulating dlPFC activation may reliably modulate explore-exploit decisions. Specific to substance use, while there has been extensive research into the neural mechanisms of addiction, it remains underexplored how individual differences in decision making serve as risk factors for increasing consumption of substances. Past investigations revealed that smokers explore less and learn faster (Merideth A. Addicott et al. 2012) and require greater cognitive control when exploring (Merideth A. Addicott et al. 2014). People with greater alcohol use tend to avoid uncertainty (L. S. Morris et al. 2016) and explore less. Brain responses may be modulated by substance use and mediated by social context (Sazhin et al. 2020). Sharing rewards with friends decreased connectivity between VS and dorsomedial prefrontal cortex (Wyngaarden et al. 2023), suggesting that social contexts are an important feature of understanding substance use decisions. Future investigation could also study the role of trends in decision making and assess whether substance users forecast future trends worse than non-substance users. Using explore-exploit dilemmas, researchers can assess how people make predictions, and whether substance users have an impaired cognitive ability to predict future outcomes.

### Conclusion

In summary, we conducted a CBMA meta-analysis of neuroimaging studies using explore-exploit tasks. We found that areas associated with executive control (dlPFC), attention (IPS, ACC), and reward (VS) are reflected in exploration and exploitation decisions. Exploration versus exploitation can be distinguished by greater activation in the dlPFC and ACC. Nonetheless, there remains substantial heterogeneity in brain responses due to task types, modulated by PCC, AI, and dmPFC activation while exploring and exploiting. Further, exploration and exploitation are associated with more similar than dissimilar patterns of activation in the AI, dmPFC, ACC, and VS. These results suggest that exploration and exploitation are not reliable opponent processes but are more of a product of the interaction of underlying physiological and psychological features guiding these decisions. Nonetheless, the finding that the dlPFC and ACC distinguish exploration and exploitation could serve as an important area of future research in cognitive neuroscience and psychopathology, as modulating these brain regions could shift how people explore and exploit.

## Figures and Tables

**Table 1:** Included studies. N=23 studies were included for the meta-analysis during explore-exploit decisions, and for the study of activation during exploration and exploitation specifically.

Paper	Program	Threshold	N	Age	Females	Task
Aberg et al., 2022	SPM	0.001	28	27.6	20	3-armed bandit
Abram et al., 2019	FSL	0.05	25	28.0	12	web-surf task
Addicott et al., 2014	SPM	0.005	22	36.0	NA	6-armed bandit
Amiez et al., 2012	AFNI	0.05	11	27.9	6	4-armed bandit
Badre et al., 2012	SPM	0.001	15	20.0	8	clock hand task
Blanchard et al., 2017	SPM	0.001	18	26.0	11	observe-bet
Chakroun et al., 2020	SPM	0.05	31	26.9	0	4-armed bandit
Daw et al., 2006	SPM	0.001	14	NA	NA	4-armed bandit
Donoso et al., 2014	SPM	0.005	40	18–26	20	4-armed bandit
Dunne et al., 2016	SPM	0.005	17	23.3	9	2-armed bandit
Howard-Jones et al., 2020	SPM	0.001	16	25.5	8	4-armed bandit
Kolling et al., 2012	FSL	0.05	20	22–32	12	foraging
Korn et al., 2018	SPM	0.001	28	23.5	13	foraging
Korn et al., 2019	SPM	0.001	24	25.0	11	foraging
Laurerio-Martinez et al., 2014	SPM	0.05	50	35.1	11	4-armed bandit
Laurerio-Martinez et al., 2015	SPM	0.05	63	35.2	11	4-armed bandit
Mobbs et al., 2013	SPM	0.05	15	25.0	10	foraging
Seymour et al., 2012	SPM	0.001	30	NA	NA	4-armed bandit
Tomov et al., 2020	SPM	0.001	31	18–35	17	2-armed bandit
Trudel et al., 2020	FSL	0.05	24	25.6	14	2-armed bandit
Wang and Voss 2014	AFNI	0.05	42	18–35	22	foraging
Wittman et al., 2016	FSL	0.05	20	21–32	8	foraging
Zacharopoulos 2018	SPM	0.001	18	18–37	21	foraging

**Table 2:** Reported contrasts. Some studies reported explore>exploit and exploit>explore contrasts, which were coded as exploration and exploitation respectively. Others reported parametrically modulated effects. The classification of those modulators as exploration and exploitation is described in the methods section. The t-threshold identified for each study is reported, which was determined by the threshold identified in the analyses conducted by each study and their respective sample size.

Paper	T -threshold	Contrasts
Aberg et al., 2022	3.6459	Explore>Exploit
Abram et al., 2019	2.0639	Parametric Modulator
Addicott et al., 2014	3.1352	Explore>Exploit
Amiez et al., 2012	2.2282	Explore>Exploit
Badre et al., 2012	4.1404	Parametric Modulator
Blanchard et al., 2017	3.9216	Explore>Exploit
Chakroun et al., 2020	2.0423	Explore>Exploit
Daw et al., 2006	4.2208	Explore>Exploit
Donoso et al., 2014	2.0227	Parametric Modulator
Dunne et al., 2016	3.252	Parametric Modulator
Howard-Jones et al., 2020	4.0728	Parametric Modulator
Kolling et al., 2012	2.093	Parametric Modulator
Korn et al., 2018	3.6896	Explore>Exploit
Korn et al., 2019	3.7921	Explore>Exploit
Laurerio-Martinez et al., 2014	2.0096	Explore>Exploit
Laurerio-Martinez et al., 2015	1.999	Explore>Exploit
Mobbs et al., 2013	2.1448	Parametric Modulator
Seymour et al., 2012	3.6594	Explore>Exploit
Tomov et al., 2020	3.6459	Parametric Modulator
Trudel et al., 2020	2.0686	Parametric Modulator
Wang and Voss 2014	1.68	Parametric Modulator
Wittman et al., 2016	2.093	Parametric Modulator
Zacharopoulos 2018	3.9651	Parametric Modulator

**Table 3:** Reported clusters of activation across meta-analyses conducted. Confirmatory analyses conducted according to the pre-registration include the explore and exploit conditions and assessing their respective conjunctions and contrasts. Exploratory analyses were conducted on n-armed bandit versus other tasks. Coordinates are reported in Montreal Neurological Institute (MNI) space.

Analysis Type	MNI coordinate	SDM-Z	P	Voxels	Cluster Location
**Confirmatory**					

All	−2,30,46	7.846	0.001	23842	Left superior frontal gyrus, medial, BA 8
	−52,8,24	4.737	0.003	1831	Left inferior frontal gyrus, opercular part, BA 44

Explore > Exploit	18,−42,−32	−2.875	0.001	1409	Middle cerebellar peduncles
	30,42,24	−3.175	0.001	919	Right middle frontal gyrus, BA 46
	−42,−38,46	−3.12	0.001	621	Left inferior parietal gyrus, BA 2
	−34,46,22	−2.988	0.012	340	Left middle frontal gyrus, BA 46
	36,−50,44	−2.515	0.024	362	Right inferior parietal gyrud, BA 40
	−6,34,24	−2.412	0.038	122	Left anterior cingulate / paracingulate gyri, BA 32

Exploit < Explore					Null
**Exploratory**					
N-armed > Other (Exploit)	54,−2,6	3.69	0.001	2041	Right rolandic operculum, BA 48
	−56,0,−16	3.77	0.001	752	Left middle temporal gyrus, BA 21
	0,−42,34	3.456	0.002	832	Left median cingulate / paracingulate gyri
	−6,−24,60	3.342	0.02	146	Left paracentral lobule, BA 4

Other > N-armed (Exploit)	6,30,40	−2.555	0.043	60	Right superior frontal gyrus, medial, BA 32
	32,26,−2	−2.643	0.042	46	Right insula, BA 47

N-armed > Other (Explore)	14,−68,44	2.191	0.021	321	Right precuneus, BA 7
	0,14,52	1.946	0.048	15	Left supplementary motor area, BA 6
	4,16,52	2.087	0.043	14	Right supplementary motor area, BA 6

Other > N-Armed (Explore)					Null

## Data Availability

Data is available on OSF at: https://osf.io/86kp9/. Thresholded and unthresholded statistical maps are located on https://neurovault.org/collections/15560/.
